# Recombination between Poliovirus and Coxsackie A Viruses of Species C: A Model of Viral Genetic Plasticity and Emergence

**DOI:** 10.3390/v3081460

**Published:** 2011-08-17

**Authors:** Nicolas Combelas, Barbara Holmblat, Marie-Line Joffret, Florence Colbère-Garapin, Francis Delpeyroux

**Affiliations:** 1 Biologie des Virus Entériques, Institut Pasteur, 75724 Paris-cedex 15, France; E-Mails: nicolas.combelas@pasteur.fr (N.C.); barbara.holmblat@pasteur.fr (B.H.); marie-line.joffret@pasteur.fr (M.-L.J.); florence.colbere-garapin@pasteur.fr (F.C.-G.); 2 INSERM U994, Institut National de Santé et de La Recherche Médicale, 75724 Paris-cedex 15, France

**Keywords:** enterovirus, poliovirus, poliomyelitis, live vaccine, vaccine-derived poliovirus, VDPV, genetic plasticity, recombination, emergence

## Abstract

Genetic recombination in RNA viruses was discovered many years ago for poliovirus (PV), an enterovirus of the *Picornaviridae* family, and studied using PV or other picornaviruses as models. Recently, recombination was shown to be a general phenomenon between different types of enteroviruses of the same species. In particular, the interest for this mechanism of genetic plasticity was renewed with the emergence of pathogenic recombinant circulating vaccine-derived polioviruses (cVDPVs), which were implicated in poliomyelitis outbreaks in several regions of the world with insufficient vaccination coverage. Most of these cVDPVs had mosaic genomes constituted of mutated poliovaccine capsid sequences and part or all of the non-structural sequences from other human enteroviruses of species C (HEV-C), in particular coxsackie A viruses. A study in Madagascar showed that recombinant cVDPVs had been co-circulating in a small population of children with many different HEV-C types. This viral ecosystem showed a surprising and extensive biodiversity associated to several types and recombinant genotypes, indicating that intertypic genetic recombination was not only a mechanism of evolution for HEV-C, but an usual mode of genetic plasticity shaping viral diversity. Results suggested that recombination may be, in conjunction with mutations, implicated in the phenotypic diversity of enterovirus strains and in the emergence of new pathogenic strains. Nevertheless, little is known about the rules and mechanisms which govern genetic exchanges between HEV-C types, as well as about the importance of intertypic recombination in generating phenotypic variation. This review summarizes our current knowledge of the mechanisms of evolution of PV, in particular recombination events leading to the emergence of recombinant cVDPVs.

## 1. Poliovirus and Poliomyelitis: Successes and Recent Hurdles in Eradication

Recombination has been known to occur in the poliovirus (PV) genome for almost fifty years [1,2] and this virus has been used as a model to investigate recombination molecular mechanisms in single stranded RNA genomes of positive polarity. Recombination was first demonstrated between various strains of this virus that belongs to the *Enterovirus* genus in the *Picornaviridae* family. There are three stable serotypes of PV, determined by the viral capsid. Natural as well as engineered heterotypic recombinants have been reported. However, it has been only recently shown that the recombination of PV with other enteroviruses can be the source of new PV strain emergence [3].

**Figure 1.**
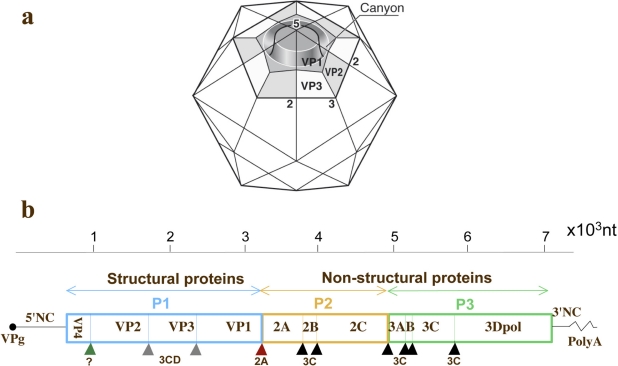
Schematic representation of the capsid and genome of poliovirus. (**a**) The icosahedral capsid of the virus is represented with the three external proteins VP1, -2 and -3 constituting a protomer. The 5-fold, 3-fold and 2-fold axis of symmetry are indicated. The canyon recognized by the cellular viral receptor CD155 is shown. (**b**) The genome is constituted of a poly-adenylated positive strand RNA genome that is covalently linked to a small viral protein VPg (also named 3B) at the 5′ terminus. The single open-reading frame is flanked by two non-coding regions (5′NC and 3′NC). The single viral polyprotein is cleaved mostly by the viral proteases 2A, 3C and 3CD (triangles indicate cleavage sites). The peptidic precursors P1, P2 and P3 are subsequently cleaved to give the different viral proteins including the capsid proteins (VP1–4) and the non-structural proteins such as the RNA polymerase 3Dpol.

Picornaviruses are small viruses composed of a non-segmented positive strand RNA genome of about 7500 nucleotides (nt) surrounded by an icosahedral capsid composed of four proteins: VP1–4 (Figure 1a). A single open-reading frame flanked by two non-coding regions (5′NC and 3′NC) characterize the viral genome (Figure 1b). Capsid proteins are encoded by the P1 genomic region and nonstructural proteins are encoded by P2–P3 regions. Viral replications cycles are almost entirely cytoplasmic. HEVs constitute a large genus of viruses classified into four species of enteroviruses (HEV-A, -B, -C and -D) and three species of rhinoviruses. PV is transmitted by the oral route, multiplies in the digestive tract, in the oropharynx and the intestine, and is excreted in stools for several weeks. Virus multiplication in the intestine is efficient but generally asymptomatic. From the digestive tract, PV reaches cervical and mesenteric lymph nodes and then establishes a viremia. Acute paralytic poliomyelitis caused by PV is associated with rare virus multiplication in motor neurons in the central nervous system (CNS), in the spinal cord, brainstem or motor cortex, in less than 1% of infections. It is generally admitted that PV enters the CNS by crossing the blood brain barrier during viremia, but a neuron-specific pathway has also been reported [4,5]. Humoral immunity plays a predominant role in the prevention and cure of PV infection.

The PV receptor (PVR) CD155 is a major determinant of PV tissue tropism, but the alpha/beta interferon also plays an important role in PV pathogenicity [6]. The interferon response limits PV movement from the periphery to the CNS [7]. Mice transgenic for the human PVR (Tg-PVR) were produced to create a convenient animal model that develops poliomyelitis following PV inoculation by various routes [8,9]. There is a very good correlation between the Tg-PVR murine and human disease in the CNS. However, these Tg-PVR mice are not a good model to mimic an oral infection by PV, and only the Tg-PVR mice that are deficient in alpha/beta interferon receptor are susceptible to oral infection by PV [10].

In 1988, the World Health Organization (WHO) established a program to eradicate poliomyelitis, based on massive vaccination campaigns with the oral polio vaccine (OPV). This vaccine is composed of live attenuated viruses of the three serotypes (Sabin strains 1–3), able to multiply to high titers only in the gastrointestinal tract. The major genetic determinants involved in the attenuation of the three OPV strains are located in the 5′NC genomic regions and are known to affect the efficiency of the Internal Ribosomes Entry Site (IRES) and genomic translation, particularly in neuronal cells [3]. In addition, codons of capsid proteins are also implicated in attenuation. OPV is known to induce a strong intestinal immune response that limits both PV replication and circulation among humans. There is also an inactivated poliovaccine (IPV) prepared with wild-type strains of the three serotypes. Both vaccines are quite efficient to protect against disease. Nevertheless, IPV is less efficient than OPV for inducing the intestinal immunity. OPV is therefore the main tool of the Poliomyelitis Eradication Initiative launched and coordinated by the WHO.

Following 20 years of massive anti-polio immunization programs, poliomyelitis due to wild-type viruses is considerably less frequent worldwide and remains endemic in only four countries (India, Pakistan, Afghanistan and Nigeria): The estimated number of polio cases dropped from 350,000 in 1988 to 715 in 2000 [11]. However, because of low vaccine coverage in some areas in countries of the developing world, the disease has not been eradicated. There were 1292 polio cases due to wild-type PV in 2010, indicating that there must have been more than 200,000 asymptomatic infected individuals. Low vaccine coverage over recent years has allowed occasional spread of wild PV strains from endemic countries to neighboring or distant countries where wild PV had disappeared [3]. In particular, there were some 600 cases of acute flaccid paralysis (AFP) and 458 cases of confirmed polio in Tajikistan and Uzbekistan in 2010, following the importation of PV from India. This constituted the first importation of polio into the WHO European Region since it was certified polio-free in 2002. Still more recently, a severe outbreak of poliomyelitis occurred in the Democratic Republic of Congo with 383 confirmed polio cases and a very high mortality rate (42% instead of the usual 5–10% rate) [12].

**Figure 2.**
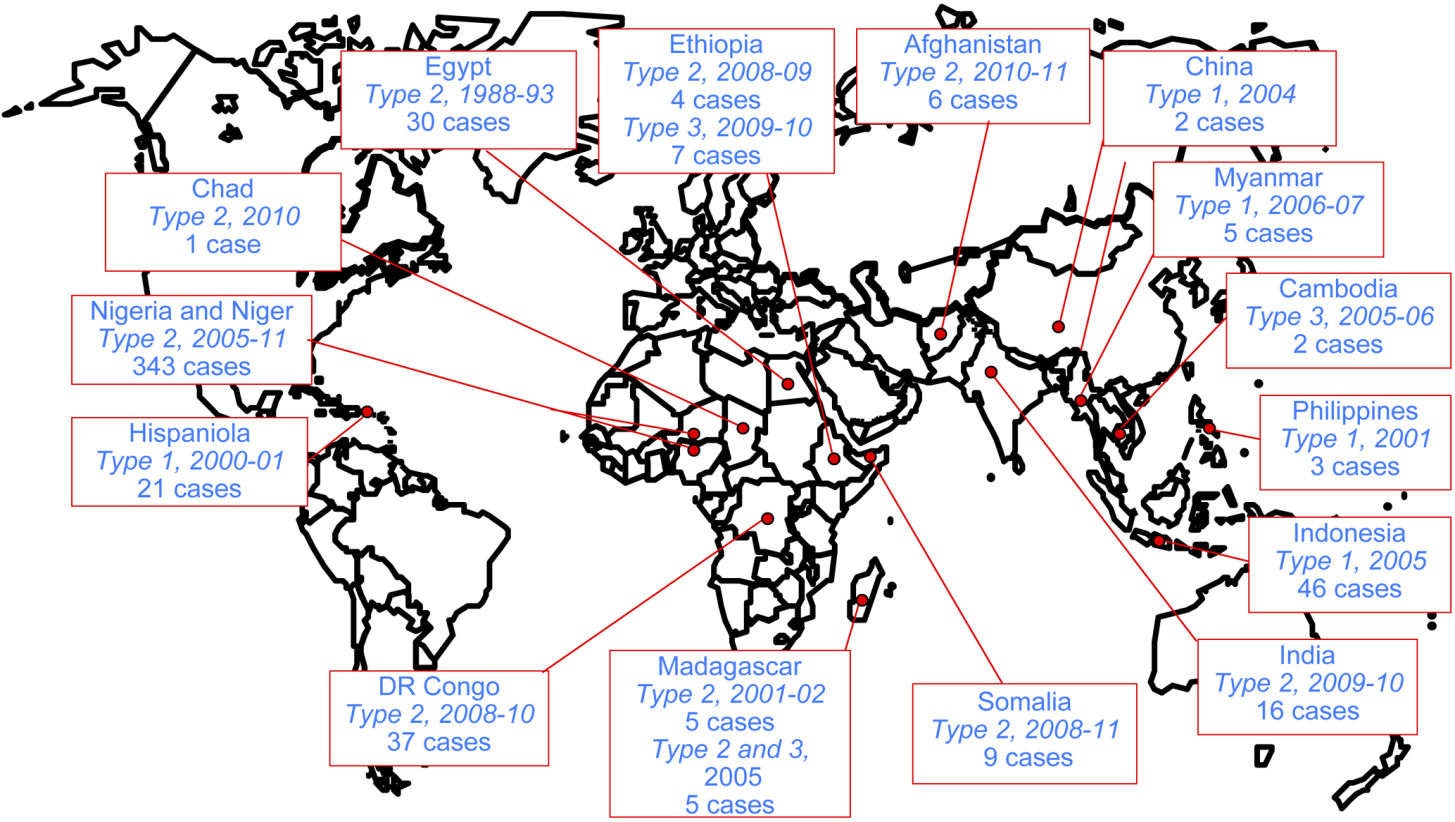
Reported poliomyelitis outbreaks due to circulating vaccine-derived polioviruses (cVDPVs) (since 1988). The country, the type of implicated cVDPVs, the year of the outbreak and the number of reported cases are indicated. Data are those available from WHO/HQ on 3 May 2011 [11].

Low vaccine coverage is also thought to allow circulation between humans, the genetic drift of OPV strains and the emergence of pathogenic, vaccine-derived polioviruses (VDPVs). These circulating VDPVs (cVDPVs) have recently caused iatrogenic epidemics of paralytic poliomyelitis in 15 regions of the world, including Madagascar in 2002 and 2005 (see below Section 4 and Figure 2). Most cVDPVs are recombinants between PV and other HEV-C, principally coxsackie A viruses (CAV). All cVDPVs have similar genomic features: the region encoding the capsid proteins is from vaccine strains, but with more than 1% nt substitutions, and some or all of the rest of the genome, particularly the region encoding nonstructural proteins, originates from other HEV-C. Thus, cVDPVs greatly complicate the implementation of surveillance and vaccination strategies aimed at eradicating poliomyelitis and PV. In addition to public health issues, the emergence of cVDPVs by recombination reveals a fascinating mechanism of variability in HEV species. Analysis of this phenomenon should provide us with new insight into viral evolution.

## 2. Poliovirus Evolution by Mutation and Recombination

The underlying mechanisms of PV evolution are mutation, homologous and non-homologous recombination, followed in all cases by selection [13,14]. Many RNA viruses replicate near the error threshold. The high mutation rate of PV is in the order of 10^−4^ errors per nt per replication cycle of the viral genome, due to the low fidelity of PV RNA polymerase 3D (3Dpol) and the absence of mismatch repair mechanism [15]. The 3Dpol error frequency was determined *in vitro* for at several sites in the viral genome, but there was no significant variation in the values [16]. However, the capsid-encoding region, with a rate of fixation of total substitutions in the order of 10^−2^ substitutions/site/year, appears less genetically stable than the region encoding non-structural proteins [17,18].

The pool of mutants differing by one or several mutations in a virus population is called quasispecies [19–21]. It has been proposed that viral populations, rather than individual variants, are the targets of evolutionary selection [22], and indeed, complementation between members in the quasispecies has been demonstrated in PV-infected mice [23]. In a wild-type viral population, some variants allow others to reach the brain after intramuscular injection, whereas a PV mutant with high fidelity polymerase has a reduced viral diversity that prevents such complementation, called cooperation [23]. A reduced cooperation capacity confers an attenuated phenotype to the mutant virus. In the presence of a defined selective pressure, the high fidelity mutant has a decreased fitness as compared to a PV with wild-type polymerase [24]. Together, these findings suggest that RNA viruses such as PV have evolved suboptimal viral polymerase fidelity to permit rapid adaptation to new environment, in particular in various host tissues [24,25]. Vignuzzi and colleagues (2008) [25] proposed to engineer attenuated virus vaccines with increased replication fidelity to improve their genetic stability and their safety.

Different molecular clocks for PV based upon the rates of substitutions into the capsid-encoding region have been calibrated. In particular, the rapid evolution of the clocks of total substitutions, synonymous substitutions and synonymous transitions can be used to estimate the dates of divergence of closely related viruses [17]. However, the lengths of successive PV infections in various hosts and that of PV chronic infection in a single immunodeficient host cannot be distinguished using these molecular clocks. Microarray analysis has been proposed to monitor the evolution of such highly divergent VDPVs [26].

Some specific amino acid alterations in VDPVs can lead to the modification of individual epitopes. However, this antigenic variability does not appear to be primarily caused by, or lead to, a significant immune evasion [27].

Recombination between viral RNA genomes is a well-known phenomenon for enteroviruses [28–31]. There are two main non-exclusive models of RNA recombination: A copy-choice, or replicative mechanism with RNA template switching during negative strand synthesis [32], and a non-replicative joining of RNA fragments [33]. It is likely that the majority of homologous recombination events occur by template switching during viral RNA replication.

V. Agol’s laboratory analyzed the primary structure of crossover regions of intertypic PV recombinants [34]. They showed that recombination sites were located in genomic regions having a potential to form secondary structures. Nonrandom distribution of the crossover sites appeared to be due to two factors: the existence of preferential sites for recombination and selection against recombinants with a lowered level of viability [35]. Using PV mutants with altered spacing of regulatory elements in the IRES, pseudorevertants suggested that RNA rearrangements (some recombinations, deletions, insertions) occur following pausing of the nascent strand, dissociation of the complex of the 3Dpol and 3′ end of the nascent strand, annealing of the nascent strand with the second template, and resumption of viral RNA synthesis [36]. The analysis of a large number of intertypic PV recombinants has also shown that cross-overs are correlated with distinctive properties of the parental RNA sequences including domains of elevated homology and of high U-A content at close proximity to recombination sites [37]. In addition, the analysis of studies of genetic recombination in a replicative cell-free system *in vitro* and in infected tissue culture cells *in vivo* have led to the observation that temperature strongly influences the loci at which cross-over between the two PV RNA strands occurs [38].

Under certain circumstances, non-replicative joining of viral RNA fragments may occur [33]. This phenomenon was demonstrated with pairs of viral RNA fragments corresponding to the 5′ NC region of the genome on the one hand, and a viral genome harboring lethal mutations, such as deletions, in the same 5′NC region. Some fragments with interruptions within the sequence of the 3Dpol were also used so that joining of the fragments was a prerequisite for the appearance of this enzyme [39]. Many recombinant PV were isolated following co-transfection of these pairs of non-infectious RNA fragments into monkey cells. Therefore, two RNA fragments could be efficiently ligated provided the 5′- and 3′-partners were in 3′-phosphate and 5′-OH forms, respectively [39]. Some recombinants appeared to be generated by multiple recombination events. Some imprecise “monstrous” but viable recombinants harboring additional sequences were genetically unstable. The additional sequences corresponded to portions of a 3′ terminal 3Dpol, 3′NC and poly A sequence. Both imprecise and precise (homologous) viable PV recombinants were obtained. There were recombination hot spots that did not preferentially map to regions of identity in the partners, but the corresponding RNA segments could be folded into secondary structures similar to known ribozyme motifs [33]. This type of recombination might have resulted from transesterification reactions. Although probably rare in nature, such events may result in the creation of novel viral genomes, and may play a pivotal role in evolution of RNA viruses.

Overall, recombination among enteroviruses is frequent [40] and Lukashev and colleagues [28] proposed that enterovirus species consist of a finite set of capsid genes responsible for different serotypes and a continuum of nonstructural protein genes that seem to evolve in a relatively independent manner. Recombination mostly occurs between members of the same species, and most often outside of the capsid encoding region [31,41]. Nevertheless, recombination in the PV capsid proteins VP1 and VP2 has been sometimes found [42–48].

## 3. Vaccine Associated Paralytic Poliomyelitis and Inter-Typic Recombinants

As mentioned above, the rate of PV evolution is extremely high and mainly due to the high frequency of nt misincorporation in RNA synthesis, [15]. This could be the cause of very rare cases (1 case per 0.2–2.5 million OPV doses) of vaccine-associated paralytic poliomyelitis (VAPP), which is clinically indistinguishable from poliomyelitis caused by wild-type PV. The first cases of VAPP appeared the year of licensure of the monovalent OPV vaccine and were associated with the Sabin 3 strain [49]. Mutated neurovirulent vaccine-derived strains were found in the gut of healthy vaccinees and in the gut and CNS of VAPP patients and most strains were intertypic vaccine/vaccine recombinants [50,51]. The underlying cause of VAPP is the genetic instability of OPV strains, as key substitutions conferring the attenuated phenotype of Sabin strains have been frequently found to be reverted in isolates from VAPP cases [52]. VAPP is most frequently associated with Sabin 3, followed by Sabin 2 [3]. The higher degree of attenuation of Sabin 1 is likely to prevent VAPP association with this strain.

Moreover, the trivalent nature of OPV provides ideal conditions for intertypic recombination between the attenuated strains of the three PV serotypes. Recombination, which is a very frequent event in PV evolution, allows the virus to eliminate adverse mutations and is likely to provide adaptation of the virus to the environment. In order to understand the evolution of vaccine strains during their replication in humans and to confirm the etiology of VAPP, numerous studies focussed on the characterization of vaccine-derived strain genomes by double restriction fragment length polymorphism assay (RFLP) [43,51,53,54]. Recombination is most often detected in Sabin 2 and Sabin 3 derived isolates and rarely in Sabin 1 [50–52,55,56]. However, sequences of Sabin type 1 origin are regularly found in S2/S1 and S3/S1 recombinant strains [57]. The reasons for this profound imbalance are still unknown but the preservation of viable secondary structures has been proposed as a possible reason for this selection [58]. The localization of certain types of recombination junctions in specific genomic regions depends on the association of strains, and certain regions of the PV genome seem to be hotspots for recombination. Most of the recombinants with a Sabin 3 capsid present recombination sites in the 2C genomic region, while recombination sites of those with a Sabin 2 capsid were preferentially located in the 3Dpol region [53,58,59]. Moreover, recent studies revealed a preferential hotspot for recombination between Sabin 3 and Sabin 2 strains, located at the 3′ end of VP1 [43,44,47,60]. Although the vast majority of recombinants present a single site of recombination, multiple sites of recombination have been identified in some isolates [43,53,54,56–59,61–63], underlining the high frequency of recombination in the PV genome, and its genetic flexibility.

In a few cases, vaccine/nonvaccines poliovirus recombinants have been found from patients with VAPP [51,61]. In these recombinants vaccine-specific segments of the Sabin virus genome have been replaced by sequences derived from wild PVs or, perhaps, from non-polio enteroviruses (NPEVs). In countries where wild-type PV still circulates, vaccine PV and vaccine/wild recombinants have been frequently isolated from the VAPP patients [54]. Recombination between wild and vaccine strains has also been identified in type 1 wild/vaccine recombinant PV sharing a 367 nt block of Sabin 1-derived sequence spanning the VP1 and 2A genes that circulated in China from 1991 to 1993 [42,62].

## 4. Vaccine-Derived Poliovirus

VDPVs are usually characterized as OPV-derived PV of the three serotypes with more than 1% nt sequence divergence from the Sabin strains in the VP1 region. OPV strains with less than 1% divergence are generally considered as OPV-like [3]. The extent of divergence is roughly proportional to the duration of viral replication or circulation since administration of the initiating OPV dose. Nucleotide substitutions in PV are known to accumulate at an overall rate of about 1% per year and the evolution rates appear to be similar for the three serotypes of PV [64–68]. This implies that VDPVs had been circulating or multiplying for more than one year, which is far beyond the normal average period of multiplication of OPV in vaccinees, rarely exceeding eight weeks [69]. It is obvious that this cutoff value of 1% sequence divergence has to be considered cautiously since OPV-derived strains with more than 0.5% sequence divergence may be considered as the product of abnormal multiplication and circulation and can be implicated in disease. In fact, rare OPV-derived type 2 OPV strains with more than 0.5% have been implicated in poliomyelitis and some of them were shown to be the source of more diverged VDPV lineages [70]. The term pre-VDPVs including type 2 OPV-like strains with 0.5–1% nt sequence divergence was recently employed [70]. Although this term presently makes sense for type 2 strains it is likely that it will possibly be applied to other serotypes if more polio cases due to type 1 and 3 OPV strains with similar percentage of nt divergence are reported.

In fact, the highest sequence divergences were reported for immunodeficiency-associated VDPV (iVDPV) isolated from immunodeficient patients who have become chronical excretors of the virus following OPV exposure and for ambiguous VDPVs (aVDPVs) that are OPV-derived strains either isolated from patients with no known immunodeficiency or not associated with an outbreak, or environmental isolates [3].

Chronic PV infection can occur following administration of OPV to individuals with primary immunodeficiencies, in particular hypogammaglobulinemia [65]. Long-term excretion of iVDPVs can last from six months to more than twenty years and is a difficult condition to treat [43,71]. Type 1 and type 2 iVDPVs were found in patients following an estimated duration of excretion of 7.6 and 19 years, respectively (10% and 18% nt sequence divergence, respectively) [71,72]. They accumulate numerous mutations and evolve toward a non-temperature sensitive and neurovirulent phenotype [73,74]. Different PV strain lineages in the same immunodeficient host, and recombination between these strains have sometimes been detected [74], as well as intertypic vaccine/vaccine recombinants [43].

Chronic PV excretors are not always identified, but highly divergent neurovirulent aVDPVs with characteristics of iVDPVs were detected in sewage when regular surveillance of the environment was performed [75–77]. Type 2 and type 3 aVDPVs with up to 13.7% sequence divergence were isolated from sewage in Slovakia and Estonia suggesting that they had been replicating in one or more individuals for approximately 10–13 years [68,78]. In addition, a type 2 aVDPV lineage has been recurrently isolated over a six year period from sewage in Israel [79,80]. Nucleotide sequence divergence values from 8.7% to 14.7% suggested that the first exposure to the vaccine strain occurred about 15 years before the last isolate uptake [80]. More recently, search for infectious PV in sewage samples in Finland led to the discovery of genetically highly divergent type 1 and 3 aVDPVs showing from 12.3% to 14.6% nt divergence from OPV strains [77]. The extent of sequence divergence suggests that the viruses had been replicating in human for more than 10 years. These type 2 and type 3 VDPVs had intertypic recombinant genomes [77]. It is likely that all these highly divergent aVDPVs had been shed from a few chronically infected individuals.

Considering the high percentage of recombinants in vaccinees [68,77,79–81], it is not surprising that certain iVDPVs and many aVDPVs have been shown to be intertypic recombinants between different types of OPV strains. To our knowledge, recombination between OPV and non-vaccine strains (either PV or other HEV-C) has never been reported in i-VDPVs or aVDPVs, even in those showing important genetic drifts. Most if not all of these VDPVs displayed the characteristics of wild PV strains. In contrast to genuine OPV strains, they can replicate at high temperature and are pathogenic in animal models [68,79–81]. They are modified in certain antigenic sites as shown by their poor reactivity with certain neutralizing monoclonal antibodies and the highly divergent ones can show lower reactivity with sera from vaccinees compared to the genuine OPV strains [68,80,81]. These VDPVs constitute a reservoir of potentially pathogenic strains that make any attempt to eradicate PVs more complex. Finding unknown PV excretors remains quite difficult and no efficient treatment has been found to cure chronically infected individuals [71,81,82]. The development of antiviral molecules capable of clearing enteroviruses from the gut of long-term excretors remains highly required.

## 5. Circulating Vaccine-Derived Polioviruses Implicated in Polio Outbreaks

**Figure 3.**
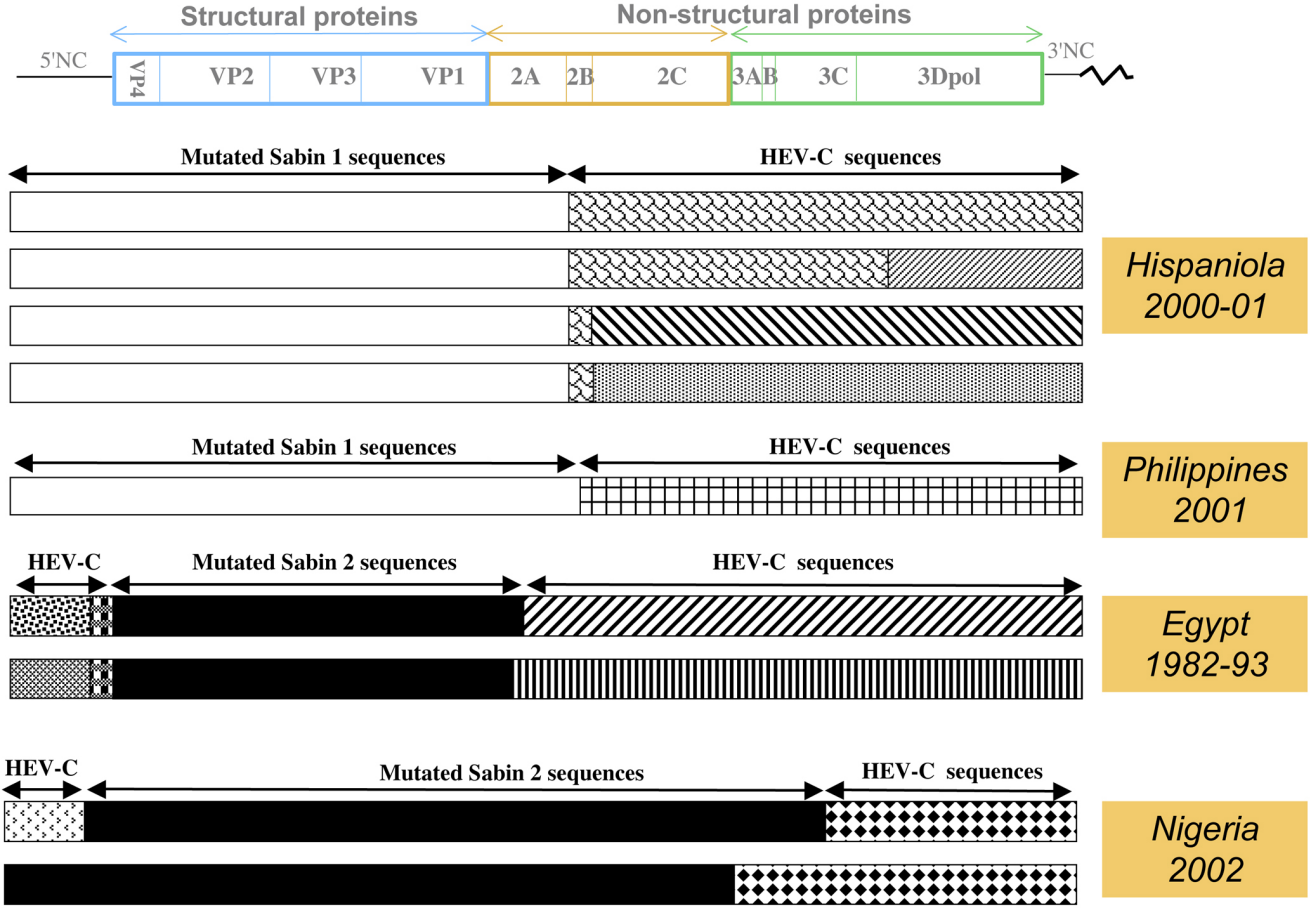
Genetic features of recombinant genomes from cVDPVs implicated in different outbreaks. A schematic view of the genetic organization of the poliovirus genome is given (see also Figure 1). The presence of vaccine-derived sequences are indicated above the cVDPV genomes (mutated Sabin 1 or 2 sequences) as well as the non-vaccine sequences derived from other HEV-Cs (HEV-C sequences). Patterns differentiate HEV-C sequences that differed significantly from each other. Data are modified from [83] (Hispaniola), [84] (Philippines), [67] (Egypt) and [85] (Nigeria).

cVDPVs are associated with sustained person-to-person transmission and have been implicated in many poliomyelitis outbreaks reported since 2000 (Figure 2). The first cVDPV outbreak to be detected occurred in 2000–2001 in Hispaniola (which is divided into the Dominican Republic and Haiti) [83]. A total of 21 confirmed cases of AFP were detected, divided into 13 cases in Dominican Republic and eight cases in Haiti, including two fatal cases. All patients were incompletely vaccinated or unvaccinated, and cases occurred in communities with very low rates of trivalent OPV vaccine coverage (from 7% to 40%). The virus isolates were unrelated to any known wild PV (<82% VP1 nt sequence identity), but closely related to the Sabin 1 OPV strain (98% VP1 nt sequence identity). According to the evolution rate within VP1 of the cVDPVs, it has been estimated that the initiating OPV dose occurred in late 1998 or early 1999. The cVDPV had recovered two of the most important biological properties of wild PV: the capacity to cause severe paralytic disease in humans and the capacity for extensive human-to-human transmission. These viruses were all recombinants presenting a 5′NC and capsid region sequences derived from Sabin 1, whereas most of the non-capsid sequences were derived from other HEV-Cs (Figure 3).

This first reported outbreak underlined that low OPV coverage carried a risk of cVDPV emergence and obliged WHO to reconsider its strategies both for polio immunization and PV surveillance. Supplemental testing requirements to increase sensitivity for detecting cVDPV were implemented, allowing another outbreak to be detected in early 2001 in the Philippines.

Three cases of acute flaccid paralysis associated with cVDPVs were reported in the Philippines during the period March 15–July 26, 2001. In contrast to the first outbreak, this outbreak appeared in an area where the vaccination rate was high (nearly 80%). The gap between different vaccination campaigns appeared as a clue for emergence of virulent cVDPV strains. The Philippines cVDPV isolates, like other cVDPVs so far, present recombinant non-capsid sequences derived from non-polio HEV-Cs [84,86]. The complete genomic sequencing indicated recombination between Sabin 1 and other HEV-Cs. Finally, the sequence relationships among the cVDPVs and Sabin 1 suggested that cVDPVs originated from an OPV dose given in 1998 or 1999.

Another outbreak, due to type 2 cVDPVs, was reported retrospectively in Egypt following the analysis of type 2 PV strains that were thought to be one of the last circulating wild type 2 strains [67]. From 1988 to 1993, 30 cases of poliomyelitis associated with PV type 2 were found in seven governorates. Recent molecular analysis of the major capsid protein VP1 revealed that the isolates were related to the Sabin 2 OPV strain and unrelated to the wild type 2 PV previously indigenous to Egypt. The sequence properties of the isolates suggested that cVDPVs emerged in Egypt with an OPV dose given in 1983 and that low OPV coverage allowed spread of infection during at least a period of a decade. Sequences analysis of both an early and a late cVDPV revealed that the 5′NC, non-structural and 3′NC genomic regions were derived from HEV-C, confirming the fact that OPV strains and HEV-Cs have frequent genetic exchanges [67].

In Madagascar, an outbreak occurred in the southern province of Toliara, between October 2001 and April 2002, where the last wild-type PV had been reported in October 1997 [87]. Five cases of AFP associated with type 2 cVDPVs occurred in two areas in 2001 and 2002. None of the patients had been fully immunized against poliomyelitis. The type 2 cVDPVs isolated from patients represented two groups depending on the area and two subgroups depending on the time of the outbreak. The urban isolates differed from the Sabin 2 strain at ∼1% of VP1 nt, whereas the rural isolates differed from Sabin 2 at ∼2.5% of VP1 nt. Sequencing of the cVDPV genomes confirmed the presence of two independent recombinant cVDPV lineages with most of the 3′ half of the genomes derived from HEV-C species [88].

**Figure 4.**
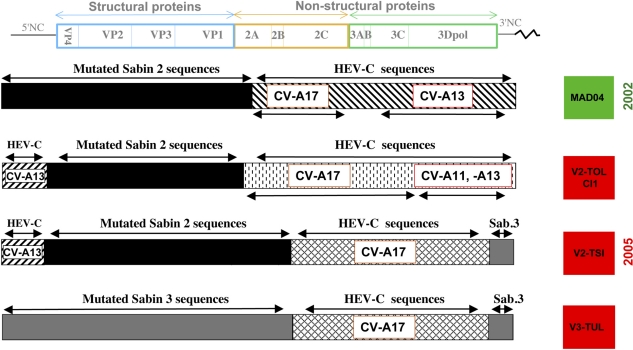
Genetic features of recombinant cVDPV genomes from Madagascar. Some cVDPVs isolated in Madagascar in 2002 and 2005 are represented as in Figure 3. Non-vaccine sequences (HEV-C sequences) showing similarity with those of coxsackie A viruses (CV-A11, -A13, -A17) isolated in the same province are indicated. Data are modified from [88–90].

Despite nationwide vaccination campaigns to interrupt circulation of cVDPVs in Madagascar, another outbreak (5 polio cases) occurred in the same province from April to August 2005. This outbreak presented particularly interesting features including that it was not only associated with type 2 cVDPVs, but also with type 3 cVDPVs. Search for viruses in the stool samples of healthy children living in close proximity to the patients showed that cVDPVs were present in 7% of children indicating that these PV were well-established in the population. VP1 and P1 nt sequences, were from 1.0% to 2.7% divergent from those of the genuine OPV strains, indicating that these strains have been circulating during about 12 to 32 months according to different transmission chains [89,90]. The cVDPV of both types presented PV/HEV-C recombinant genomes, confirming that cVDPVs are associated with the co-circulation of HEV-Cs and with genetic exchanges between both partners. Despite the fact that these cVDPVs were isolated in different and distant districts of the same province of Madagascar they shared common genetic features (Figure 4). The type 2 cVDPV genomes shared similar 5′ halves constituted of the non-vaccine HEV-C sequences in the 5′NC region and identical recombination sites with the neighboring OPV sequences. In addition, some type 2 and the type 3 cVDPV isolates shared the same 3′ end that was made of non-vaccine HEV-C and Sabin 3 sequences with identical recombination sites. All these results indicated an intense circulation of enterovirus strains and a rapid evolution between PV and HEV-C, involving multiple rounds of intratypic and intertypic recombination in a short period of time and in a restricted geographic area [89,90].

Since the first reports of polio outbreaks, new epidemics due to the circulation of cVDPVs were reported (see Figure 2 and [11]). Most of these outbreaks implicated only a limited number of up to a few dozen cases. However, a severe outbreak due to type 2 cVDPVs was rife in Nigeria from 2005 for more than six years with more than 300 cases of poliomyelitis [70,91,92]. In general, the studied VP1 nt sequences of cVDPV isolates have diverged from parental OPV strains by only 1–3.5%, which indicates circulation times of no longer than 1–4 years and reversion of the attenuated phenotype of Sabin strains was observed [83,84,87,93,94]. However, nucleotide divergence of up to 6% could be found recently in Nigeria [95]. It is striking that the cVDPVs implicated in most outbreaks mentioned above were due to recombinant strains which were the product of genetic exchanges between OPV strains and other HEV-Cs (Figures 3 and 4 and Table 1) [3,70,89,96] including the recombinant type 2 cVDPVs implicated in recent polio outbreaks in India and in Democratic Republic of Congo [95,97]. Up to now only one polio outbreak with two cases of disease occurring in Guizhou Province, China, was due to a non-recombinant cVDPV Sabin 1 lineage with 1.0%–1.2% nt divergence [98].

Although all the cVDPV outbreaks occurred in low-income countries, clear indication of circulation of vaccine-derived viruses could also be found in industrialized countries with temperate climates and middle population density if the following conditions are present: Wide immunity gap, number and density of immune susceptible persons, high birth rate and deficiencies in hygiene/sanitation. Despite high global vaccine coverage with OPV, a type 1 VDPV has been isolated from one AFP case and seven healthy children (contacts) living in a small town in Romania in a minority with low vaccine-coverage and bad hygiene conditions [55]. The isolated strain exhibited a tripartite intertypic recombinant genome Sabin 1/ Sabin 2/ Sabin 1 derived from a common ancestor strain, with 1.2% nt substitutions in the VP1 capsid protein coding region, indicating a circulation time of about 14 months. Moreover, an imported type 1 VDPV differing by 2.3% nt sequence from the genuine Sabin 1 strain was found in many children living in an undervaccinated community in Minnesota [99]. Suspicion of long-term circulation was also inferred from the analysis of divergent OPV strains isolated from patients with AFP in the Russian Federation [26,52,100] or from non-immunized populations in Byelorussia [101]. Therefore, even in developed countries, small populations with low vaccine coverage, living in globally well-vaccinated countries can be the origin of cVDPV emergence and circulation, reaffirming the importance of a well-organized AFP surveillance program.

## 6. Genetic Features of Recombinant cVDPV Genomes

All recombinant cVDPV genomes described so far had kept at least the entire region encoding the capsid proteins of OPV strains (with more than 1% nt substitutions), and some or all of the rest of the genome, particularly the region encoding non-structural proteins, originates from other HEV-Cs [67,83,84,88–90]. Most of them showed the 3′-half of their genome derived from non-OPV sequences with recombination sites located in proteins 2A or 2B [67,83,84,88]. Nevertheless, recombination sites could be found elsewhere in the P2 or the P3 region [89,90]. In addition, many type 2 VDPV lineages displayed a great part of their 5′NC region derived from non-OPV HEV-Cs. In this case some of them had only kept the region encoding the capsid proteins from the genuine OPV strains (Figures 3 and 4). More complex genomic structures were found in Madagascar during the 2005 outbreak. Type 2 cVDPV lineages showed a mosaic quadripartite recombinant genome constituted of non-OPV sequences in the 5′NC and in the P2/P3 regions, flanking the type 2 OPV capsid sequences and, moreover, type 3 OPV sequences ended the 3′ termini of the genome (Figure 4) [89].

When sequenced, all major attenuation determinants located in the 5′NC OPV genomic regions and known to affect the efficiency of genomic translation were found to have reverted to wild type nt. These determinants are located at nt positions 480, 481 and 472 of the Sabin 1, 2 and 3 OPV strains, respectively. In addition, in type 1 cVDPVs, codons implicated in attenuation were sometimes mutated in capsid proteins, in particular at residues 65 of VP4, 225 of VP3 and 106 of VP1 [83,84,102]. Residues 143 of VP1 and 91 of VP3 implicated in the attenuation of the type 2 and 3 OPV strains, respectively, were systematically mutated in the corresponding cVDPVs [67,88–90].

## 7. Recombination Partners of PV in cVDPVs

The finding that some recombinant cVDPV genomes have kept only the capsid genomic regions from the genuine OPV strains [54,61,67,89,90] indicated that all the other parts of the genome can be shared by other HEV-Cs. The enterovirus capsid, rarely subject to recombination [31], appears as the main viral structure that determines virus types. The type of wild PV and other HEV-Cs refers thus to the structure and sequences of the capsid for a given virus. Actually, a good correlation was found between the nt sequences of the capsid protein VP1 and the serotype of known enteroviruses [103,104]. Comparing nt VP1 sequences of a HEV with those of prototype HEV strains has become the main method to identify the type of an isolate.

HEV-Cs were the suspected partners of recombination with OPV strains, because of the higher similarity of their genomic sequences with that of PV, as compared to those of other HEVs belonging to the HEV-A, HEV-B or HEV-D species [105]. In fact, vaccine-derived recombinants with non-vaccine sequences were isolated in several countries (Romania, Egypt, Nigeria, Democratic republic of Congo, India) in which wild type strains were still circulating [54,61,67,85]. In these cases, recombination between OPV and wild-type PV strains could be questioned. However, OPV/HEV-C recombinants were also isolated in countries (Hispaniola, Philippines, Cambodia and Madagascar) in which wild PVs had been eradicated [83,84,88,89,94]. Therefore, the hypothesis of recombination between OPV and co-circulating non-PV HEV-Cs was put forward.

This hypothesis was confirmed in Cambodia, in which a Sabin 3-/HEV-C recombinant (Cambodia 02) was isolated from an AFP case in 2002 [94]. An attempt to identify the putative recombinant counterpart was done by partially sequencing non-PV HEV-Cs which had been isolated from patients with AFP between 1999 and 2003 in the country. The results indicated that parts of the P2 and the 3Dpol genomic regions of the strain Cambodia 02 were closely related to homologous sequences of CAV strains of type 17 and 13 (CV-A17 and CV-A13), respectively. These results suggested that the P2 and the 3DPol genomic regions of the Cambodian VDPV were derived from a HEV-C strain genetically related to indigenous CV-A17 and CV-A13 strains circulating in Cambodia. Concerning the type 2 recombinant cVDPVs isolated in Madagascar, the putative parents of their non-PV sequences were searched for in HEV isolates present in stool samples from healthy children [88]. These samples were collected in the district where the AFP cases were reported in 2002, in villages located in a small area, a few kilometers away from each other. 21% of the samples were positive for HEVs and 80% of these HEVs belonged to the HEV-C species including six different types (CV-A11, CV-A13, CV-A17, CV-A20, CV-A24 and HEV99) [88,90]. Partial sequencing suggested that parts of the P2 and 3Dpol genomic regions of the cVDPVs isolated in 2002 were derived from HEV-C strains genetically related to Madagascar CV-A17 and CV-A13 strains, respectively. Non-PV sequences present in the cVDPV lineages isolated in 2005 were also related to sequences present in local CV-A13, CV-A17 and possibly CV-A11 (Figure 4) [90].

These results indicated that most of the genomes of the cVDPVs were complex mosaic genomes with sequences derived through genetic recombination from different OPV and CAV strains. CV-A13 and CV-A17 appeared from these studies as the preferred partners for recombination with OPV strains.

## 8. PV, a Member of a Dense and Diversified HEV-C Ecosystem

In fact, the search for the non-PV partners that recombined with OPV strains to give cVDPVs in Cambodia and in Madagascar led to the discovery of many co-circulating non-PV HEV-Cs [88,94,107]. In both countries the percentages of HEVs belonging to species C were high (38% and 80%, respectively). Six to seven different HEV-C types were found each year in Cambodia from 1999 to 2003. A similar variety of HEV-C types co-circulating with recombinant cVDPVs was also found in Madagascar from samples collected over a few days, in a small area. A large variety of recombinant genotypes was found, increasing the diversity of this dense HEV-C ecosystem [88,108]. It is likely that PV has been always an active member of this ecosystem. OPV strains do have the natural propensity to interact with the other members of the HEV-C species when they come into contact. The presence of multiple HEV-C types is probably due to particularly favorable conditions (climate, poor sanitation, human genetics, *etc.*). Another non-exclusive hypothesis is that genetic and functional interactions among these different types contribute to maintain this viral diversity.

Among the different co-circulating HEV-C types that have been identified so far in Cambodia and in Madagascar, only PV is known to be a major human pathogen. CV-A1, CV-A11, -13, -17, -20 are not known to be implicated in particular severe disease outbreaks and may be considered as poorly- or non-pathogenic viruses [109]. Only CV-A21 and CV-A24 have been reported to be implicated in common cold outbreaks. CV-A24 is known to be the causative agent of important hemorrhagic conjunctivitis outbreaks [109]. PV appears thus as the “black sheep” of a much more quiet “viral tribe”. It differs also from the other HEV-C members by many biological properties including binding on a specific cellular receptor CD155 whereas most of the others are thought to bind to ICAM-1. This HEV-C ecosystem gathers thus very different viruses even though they belong to the same species.

## 9. Impact of Non-PV Sequences on the Characteristics of cVDPVs—*in vitro*-Made Recombinants

Most of the cVDPVs that have been described so far share phenotypic characteristics including the capacity to induce poliomyelitis in humans and in transgenic mice expressing CD155, the human cellular receptor for PV [67,83,84,88]. In addition, in contrast to the genuine OPV strains, cVDPVs can efficiently multiply at supraoptimal temperature. These characteristics correlate with the modification of genetic determinants implicated in attenuation and temperature sensitivity. This could be done through mutation or through recombination with non-OPV sequences. Actually, no phenotypic character that can be evaluated in laboratories can distinguish cVDPVs from wild counterparts.

It has recently been reported that, in Nigeria no significant differences could be found in the clinical severity and attack rate of paralysis among the cases due to cVDPVs and those due to wild PVs. Most of the cVDPVs that have been studied in details and in some cases with batteries of neutralizing monoclonal antibodies, showed modifications in some neutralizing antigenic sites [66,83,84,88]. However, these antigenic modifications, resulting from mutations in the capsid genomic region, have no dramatic influence on the efficiency of the OPV since OPV vaccine responses organized following cVDPV outbreaks were capable of getting rid of the implicated viruses [110].

Genetic recombination is a common evolution mechanism that is thought to contribute at least to the repair of deleterious mutations in genomes [13]. However the role of genetic exchanges in the characteristics of OPV/HEV-C recombinant is still unclear. The characteristics of the PV genome (small size, non-segmented, positive polarity, infectious RNA) facilitated genetic engineering and the construction of chimeric PV. This allowed to locate viral determinants such as those involved in neurovirulence/attenuation, temperature sensitivity, host cell tropism, and to investigate the mechanisms of recombination. It also allowed the impact of recombination between PV and other HEVs to be investigated.

The genetic exchanges between a wild type 1 PV (PV1) and CAV, in particular CV-A20, have been studied with engineered chimeras [111]. The genetic structure resembling that of cVDPV, with a PV capsid, consistently survived much better than did their reciprocal counterparts with a CAV capsid. Similar results were obtained with other serotypes, indicating that genetic recombination between PV and CAV showed a parent-specific asymmetry in compatibility [111]. In addition, the inter-virus genetic distance of parents modulated compatibility. The recombination frequency between PV1 and CV-A20 was calculated to be 10^−6^, similar to frequencies observed between heterotypic PV strains. The cross-overs mapped to the P2 region encoding nonstructural proteins. Interestingly, the PV1/CV-A20 recombinants were nearly as neurovirulent in Tg-PVR mice as wild PV1 [111]. This suggested that some CAVs can be recombination partners of PV in nature. More recently it was shown that a direct interaction between the non-structural protein 2C and the capsid protein VP3 is required for enterovirus morphogenesis [112]. A non-functional interaction between the protein VP3 of some CAVs and the protein 2C of PV could explain the defect in multiplication of CAV/PV recombinants [111,112] and their apparent absence in nature [88].

To investigate the impact of genetic exchanges between PV and HEV-C species on the characteristics of recombinant viruses, Riquet and colleagues generated chimeric viruses with various cross-over regions between one type 2 cVDPV and the original vaccine Sabin 2 strain [113]. Protease 2A from cVDPV played only a minor role or no role at all in the pathogenicity of these *in vitro* made recombinants. Results indicated that the other HEV-C sequences present in this cVDPV contribute to its characteristics, including pathogenicity, suggesting that PV/CAV recombination may favor the emergence of cVDPVs [113].

More recently, Jegouic and colleagues investigated whether CV-A17 can act as a recombinant partner of PV [114]. They generated recombinant constructs combining the genetic material of a CV-A17 isolate from Madagascar with that of the type 2 vaccine strain and that of a type 2 cVDPV. PV/CV-A17 recombinants were all viable. The recombinant in which the 3′ half of the vaccine strain genome had been replaced by that of the CV-A17 genome yielded larger plaques and was less temperature sensitive than its parental strains. It was almost as neurovirulent as the cVDPV in transgenic mice expressing the PV cellular receptor gene. This study showed that a CV-A17 isolate co-circulating with a PV/HEV-C recombinant cVDPV can be a recombination partner for PV. These results also suggested that recombination can, at least in some cases, have a direct effect on the key phenotypic characteristics of recombinants, such as replication level, and may thus favor the emergence of pathogenic cVDPVs.

## 10. A Model of Genetic Plasticity and Emergence

Complex vaccine-derived multi-recombinant PVs including a penta-recombinant PV implicating the three serotypes have been reported [48,58]. Multi-recombinants of OPV strains with wild PVs were also described [42,54,61,62]. In addition, genetic exchanges between wild PVs were reported [62,115]. Actually, different complex mosaic genomes implicating different serotypes of OPV strains and different genomic fragments related to different CAV types were found in Madagascar in a short period of time [89,90]. Together these data show that a given PV type can evolve rapidly and intensively through recombination by multiplying and circulating in close interaction with other PV or HEV-C types. With regard to the biodiversity of HEV-Cs found in a single district of Madagascar, and to the rapid spread, evolution and diversification of the recombinant cVDPV genotypes in a short period of time, it is tempting to propose that, in this kind of ecosystem, intratypic as well as intertypic recombination between HEVCs is a permanent and usual mechanism of genetic plasticity that in addition to mutations generates genetic and phenotypic viral diversity.

Studies with engineered PV/CAV recombinants have shown that recombination can affect—or not—the multiplication of recombinant viruses [111–114]. Similarly, experiments consisting in evaluating the pathogenicity of different *in vitro*-made PV/CAV recombinants in mice suggested that recombination can be neutral or can modulate the pathogenicity of recombinants [111,113,114]. These results suggest that the genetic plasticity associated with recombination events could either favor or affect the fitness of these viruses and their capacity to circulate in the human population and, incidentally, could make them more or less pathogenic.

Since most cVDPVs implicated in polio outbreaks are OPV/HEV-Cs recombinants, it is likely that the genetic interaction between OPV strains and other HEV-Cs provides the virus with another way to increase its capacities to multiply, to circulate and eventually to emerge as a pathogenic and epidemic strain. That the main genetic determinant affecting translation and neurovirulence of OPV strains in the 5′NC genomic region is reverted either by mutation or by recombination in cVDPVs lend support to this idea [67,89,90]. We cannot completely exclude that viral interactions via intertypic recombination is a neutral consequence of the simultaneous presence of multiple HEV-C types. However, this hypothesis seems nowadays unlikely.

## 11. Concluding Remarks

The global public health program consisting of vaccination of the human population with OPV has been quite successful. However, the emergence of cVDPVs is one of the major issues threatening the benefit of the eradication program. In fact, the spreading of a huge amount of man-made attenuated vaccine virus in the human population allowed us to discover an unknown interaction between PV and the members of its natural ecological niche. This discovery is of tremendous importance in terms of medical as well as basic virology and underlines the interest to keep close contact between basic and medical sciences, in particular when viral human pathogens are concerned.

Many questions still remain concerning the parameters and interactions contributing to the maintenance and evolution of the different HEV-C types in the same ecosystem and niche. The mechanisms allowing frequent co-infection of cells by different virus types, those contributing to the recombination process, and the factors implicated in the likely subsequent selection of certain recombinants are still poorly known.

A better understanding of the environmental and viral factors shaping HEV-C genetic diversity and contributing to the emergence of cVDPVs will certainly allow to improve viral surveillance and vaccination strategies and to prevent outbreaks due to vaccine-derived recombinant polioviruses.

## Figures and Tables

**Table 1.** Recombinant features of circulating vaccine-derived polioviruses (cVDPVs) implicated in polio outbreaks. The type of capsid is indicated as well as the presence of non-vaccine sequences (HEV-C) in at least part of the non-structural genomic regions. Niger and Chad cVDPVs are linked to the Nigeria outbreak.

**Country**	**Number of Cases**	**Year(s) of Outbreak**	**cVDPV capsid/non capsid coding region**	**References**
Indonesia	46	2005	OPV type 1 / HEV-C	[96]
China	2	2004	OPV type 1	[98]
Philippines	3	2001	OPV type 1 / HEV-C	[84]
DOR/Haiti	21	2000–01	OPV type 1 / HEV-C	[83]
Egypt	30	1988–1993	OPV type 2 / HEV-C	[67]
Nigeria	338	2005–2011	OPV type 2 / HEV-C	[85]
Chad	1	2010	OPV type 2 / HEV-C	[95]
DR Congo	37	2008–10	OPV type 2 / HEV-C	[95]
Niger	5	2006–2010	OPV type 2 / HEV-C	[95]
Cambodia	3	2005–06	OPV type 3 / HEV-C	[106]
India	16	2009–10	OPV type 2 / HEV-C	[97]
Madagascar	10	2001–02	OPV type 2 / HEV-C	[88]
		2005	OPV type 3 / HEV-C	[90]
